# Child mortality from solid-fuel use in India: a nationally-representative case-control study

**DOI:** 10.1186/1471-2458-10-491

**Published:** 2010-08-17

**Authors:** Diego G Bassani, Prabhat Jha, Neeraj Dhingra, Rajesh Kumar

**Affiliations:** 1 Centre for Global Health Research (CGHR), Keenan Research Centre, Li Ka Shing Knowledge Institute, St. Michael's Hospital, Toronto, Canada; 2 Dalla Lana School of Public Health, University of Toronto, Toronto, Canada; 3 Child Health Evaluative Sciences, Hospital for Sick Children, Toronto, Canada; 4 Najafgarh Rural Health Training Centre, Ministry of Health, Government of India, New Delhi, India; 5 School of Public Health, PGIMER, Chandigarh, India

## Abstract

**Background:**

Most households in low and middle income countries, including in India, use solid fuels (coal/coke/lignite, firewood, dung, and crop residue) for cooking and heating. Such fuels increase child mortality, chiefly from acute respiratory infection. There are, however, few direct estimates of the impact of solid fuel on child mortality in India.

**Methods:**

We compared household solid fuel use in 1998 between 6790 child deaths, from all causes, in the previous year and 609 601 living children living in 1.1 million nationally-representative homes in India. Analyses were stratified by child's gender, age (neonatal, post-neonatal, 1-4 years) and colder versus warmer states. We also examined the association of solid fuel to non-fatal pneumonias.

**Results:**

Solid fuel use was very common (87% in households with child deaths and 77% in households with living children). After adjustment for demographic factors and living conditions, solid-fuel use significantly increase child deaths at ages 1-4 (prevalence ratio (PR) boys: 1.30, 95%CI 1.08-1.56; girls: 1.33, 95%CI 1.12-1.58). More girls than boys died from exposure to solid fuels. Solid fuel use was also associated with non-fatal pneumonia (boys: PR 1.54 95%CI 1.01-2.35; girls: PR 1.94 95%CI 1.13-3.33).

**Conclusions:**

Child mortality risks, from all causes, due to solid fuel exposure were lower than previously, but as exposure was common solid, fuel caused 6% of all deaths at ages 0-4, 20% of deaths at ages 1-4 or 128 000 child deaths in India in 2004. Solid fuel use has declined only modestly in the last decade. Aside from reducing exposure, complementary strategies such as immunization and treatment could also reduce child mortality from acute respiratory infections.

## Background

**Figure 1:**
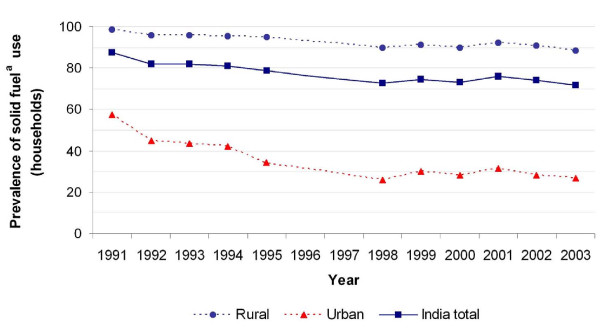
**Percentage of households using solid fuels **^**a **^**as major source for cooking and heating from 1993 to 2003, by rural and urban areas **Footnote: ^a ^Solid fuels: coal/coke/lignite, fire wood, dung and crop residue. Year: 1991 Source: Census of India, 2001 Year: 1992 Source: Household Consumer Expenditure and Employment Situation in India, NSS Report No. 397, 48th Round Year: 1993 Source: Housing Conditions in India, NSS Report No. 429, 49th Round Year: 1994 Source: Energy Used by Indian Households, NSS Report No. 410/2, 50th Round Year: 1995 Source: Household Consumer Expenditure and Employment Situation in India, NSS Report No. 436, 51st Round Year: 1998 Source: National Family Health Survey (NFHS-2), India Year: 1999 Source: Energy Used by Indian Households, NSS Report No. 464, 55th Round Year: 2000 Source: Household Consumer Expenditure and Employment-Unemployment Situation in India, NSS Report No. 476, 56th Round Year: 2001 Source: Census of India, 2001 Year: 2002 Source: Housing Condition in India (Household Amenities and Other Characteristics), NSS Report No. 489, 58th Round Year: 2003 Source: Household Consumer Expenditure and Employment-Unemployment Situation in India, NSS Report No. 490, 59th Round

Half of the world's population and two thirds of India's population use solid fuels (coal/coke/lignite, firewood, dung, and crop residue) for cooking and heating (Figure 1) [1]. Indirect estimates suggest that exposure to indoor smoke from solid fuels is responsible for over half of the 1.8 million annual worldwide child deaths from acute lower respiratory infections [2], making it the largest environmental factor causing ill health and deaths among children globally [1,3-7]. Children under age 5 spend many hours breathing indoor smoke, especially during their first year of life - when they are carried by their mothers. Such exposure to indoor smoke that contains several health-hazardous substances and particles is especially problematic in this early life phase where airways are still being developed and therefore are much more vulnerable. Although it is estimated that close to 60% of all indoor air pollution attributable deaths occur among children under age five, direct research on the effects of solid-fuel use on child survival has been limited [8] and the indirect mortality estimates are based on small studies with wide ranges of risks and include few studies from India [9].

Direct measurement of the impact of solid fuel use on child survival has to take into account several risk factors that increase mortality and are associated with solid-fuel use, chiefly as a consequence of poverty, such as living conditions, sanitation and access to water [9,10]. Most studies in low and middle income countries do not include a large enough sample to study the impact of solid-fuel use on child mortality with adjustment for these factors [11]. We report here the results of a large nationally-representative population-based case-control study of the association between solid-fuel use and all-cause child mortality in 1.1 million households in India in 1998.

## Methods

This case-control study compares the prevalence of solid-fuel use between households where children under age 5 died and households of living controls within the Special Fertility and Mortality Survey (SFMS) - a nationally-representative household survey conducted in 1998 to obtain fertility history of ever-married women in India [12]. The SFMS was undertaken by the Registrar General of India in the sample units of the Sample Registration System (SRS) - an ongoing demographic survey that annually estimates fertility and mortality indicators for India's major states and nationally. The sample frame covers 5.9 million people, living in 1.1 million households in 35 states/territories of India. The 6671 (4436 rural and 2235 urban) SRS units were selected using stratified simple random sampling based the 1991 Census. Each unit comprises about 150 households and 900 people. The survey was done in February 1998 and collected information about the village, household, living individuals, women's fertility history and deaths that occurred in the household in the previous calendar year. Surveys were implemented by 700 trained supervisors who conduct the SRS half-yearly surveys. Household residents gave oral consent and followed confidentiality and ethical guidelines as per ongoing census activities. Further details of the SRS and SFMS sample and field methods are published elsewhere [12].

Cases were all children under the age of 5 years in the SFMS households that died between 1/1/1997-31/12/1997. Cases were stratified by gender and sub-divided into: post-neonatal (death between 28 days post partum and the first year of life, henceforth *post-neonatal deaths*) and deaths between ages 1-4 years. Living children below 1 year of age were included as controls for post-neonatal deaths and living children ages 1-4 years were the controls for deaths at ages 1-4 years.

To study the association of solid-fuel use and child deaths, we explored the ratio of the prevalence of solid-fuel use in the household via the following comparisons:

(a) deaths at ages 1-4 years compared to living children of same age;

(b) post-neonatal deaths compared to living children below 1 year of age and;

(c) deaths at ages 1-4 years compared to neonatal deaths. Neonatal deaths were selected as control in this sub-analysis because the biological plausibility for the occurrence of neonatal deaths in consequence of exposure to solid fuels is lower (due to the short period of direct exposure). At the same time, children who died during the neonatal period share more characteristics with children that died later (cases - child deaths) than living controls. Therefore, unmeasured confounding is expected to be minimized in this analysis as is residual confounding - if present.

A sub-analysis compared solid-fuel use among deaths at ages 1-4 years occurring during the winter months (December, January and February) in the states with lower than average winter temperatures to solid-fuel use among:

(a) living children of the same age and;

(b) children of the same age that died in non-winter months.

In a secondary analysis, living children ages 1-4 years who reported pneumonia in the three months preceding data collection (December 1997, January/February 1998) were selected as cases, and living children of same age who did not report pneumonia were selected as controls.

Fuels used for cooking and heating in the household were classified into: (i) non-solid fuels: kerosene, gas/natural gas, electricity and biogas; (ii) solid fuels: coal/coke/lignite, firewood, dung, and crop residue. Kitchen and cooking areas were directly observed by the interviewer and classified as separated or non-separated from living areas. The type of fuel used for cooking/heating and the type of kitchen were combined in a three-level variable: Non-solid fuels; solid fuels in separate kitchen and solid fuels in non-separate kitchen. Information about exhaustion systems/chimneys was not collected. Field interviewers directly observed the material used in the construction of roof, walls, and floor of the house and categorized according to the quality of the material as high-quality, mixed and low-quality. Other socio-demographic variables included: location of the household (rural or urban), education of the head of the household (illiterate, literate/primary education, middle education or above), religion (Hindu, Muslim, and other) and the number of siblings (continuous). Source of drinking water was categorized as clean (tap, hand-pump, and tube-well/bore-well) and non-clean (open well, tank/pond, river/lake/spring). Latrine was categorized as: inside the house with regular flush, inside the house with dry/chemical flush, outside the house, and absent.

Analyses were performed using Stata SE 10.0 [13]. Prevalence Ratios (PR) were estimated through Poisson regression through an hierarchical multilevel [14]. The 95% confidence intervals (95%CI) for the estimates were calculated using a variance correction factor for sampling unit and family clustering. Confounders were addressed in the analysis section by stratification (gender) and adjustment (other variables). Analysis was restricted to individuals with complete data on solid-fuel use. The maximum frequency of missing values observed for any variable was 3.5%. Exclusion of the 1612 households with both living and deceased children (0.5% of all households) did not alter the results (data not shown). The 1998 risks were projected onto the 2004 World Health Organization (WHO) totals of child deaths in India [2,15].

## Results

We included 6790 deaths divided into post-neonatal deaths (1436 boys and 1790 girls), and deaths at ages 1-4 years (1568 boys and 1996 girls). A total of 609 601 living children are included as controls: 130 402 aged less than one year (68 529 boys and 61 873 girls), and 479 199 aged 1-4 years (250 883 boys and 228 316 girls). The 5590 neonatal deaths (3077 boys and 2513 girls) served as controls in the case-only analysis. Deaths at ages 1-4 years among boys and girls were more common in rural areas, in households with lower education, and these children had more siblings than living controls (Table 1). Compared to living children ages 1-4 years, a larger proportion of deaths at ages 1-4 years came from houses built with low-quality materials (boys: PR 1.64 95%CI 1.44-1.87; girls: PR 1.37 95%CI 1.23-1.54). Boys that died at ages 1-4 years had less access to clean water than living boys of the same age (PR 1.19; 95%CI 1.05-1.34). No access to a latrine was more common among girls that died at ages 1-4 years than among living girls of same age (PR 1.74; 95%CI 1.47-2.06). Solid fuels were used in about 87% of the households where a child died at ages 1-4 years, versus about 77% of the households with living children ages 1-4 years. Solid-fuel use in non-separate kitchens/cooking areas was also more common in households with deaths at ages 1-4 years (up to 50%) than in households with living children of the same age (about 37%).

**Table 1:** Characteristics of child deaths (between ages 1 to 4 years) and living controls (living children, ages 1 to 4), India 1998-1999.

	Boys							Girls						
	
	Child Deaths/Living Controls							Child Deaths/Living Controls						
	(n = 1,568)		(n = 250,883)					(n = 1,996)		(n = 228,316)				
	n		%		PR*	(95% CI)		n		%		PR*	(95% CI)	
**Household demographics**														
**Rural/Urban**														
Urban	260/	56,800	16.6/	22.6	1.00			307/	51,340	15.4/	22.5	1.00		
Rural	1,308/	194,083	83.4/	77.4	**1.30**	**(1.04-**	**1.51)**	1,689/	176,976	84.6/	77.5	**1.22**	**(1.04-**	**1.44)**
**Education Head of household**														
Middle education or above	212/	65,176	13.6/	26.2	1.00			282/	58,062	14.3/	25.6	1.00		
Literate/Primary education	394/	73,783	25.3/	29.6	**1.40**	**(1.15-**	**1.70)**	499/	67,641	25.3/	29.8	**1.21**	**(1.02-**	**1.44)**
Illiterate	952/	110,151	61.1/	44.2	**2.04**	**(1.70-**	**2.45)**	1,194/	101,116	60.5/	44.6	**1.67**	**(1.43-**	**1.95)**
**Religion**														
Hindu	1,223/	196,205	78.1/	78.3	1.00			1,585/	177,719	79.5/	77.9	1.00		
Muslim	247/	36,231	15.8/	14.5	1.03	(0.90-	1.19)	315/	33,887	15.8/	14.9	1.05	(0.92-	1.18)
Other	96/	18,198	6.1/	7.3	1.04	(0.84-	1.28)	95/	16,471	4.8/	7.2	0.84	(0.68-	1.04)
**Living conditions**														
**Number of siblings**														
0 to 1	218/	41,313	14.1/	16.5	1.00			234/	37,159	11.9/	16.3	1.00		
2 or 3	516/	105,196	33.3/	42.0	1.00	(0.81-	1.24)	658/	94,592	33.5/	41.5	1.18	(0.96-	1.44)
4 or more	816/	103,690	52.7/	41.4	**1.44**	**(1.18-**	**1.76)**	1,073/	95,989	54.6/	42.2	**1.73**	**(1.43-**	**2.10)**
**Type of house**^**a**^														
Pucca	404/	100,348	25.8/	40.0	1.00			529/	90,197	26.5/	39.6	1.00		
Semi-pucca	288/	48,809	18.4/	19.5	**1.19**	**(1.02-**	**1.40)**	388/	44,067	19.5/	19.3	**1.15**	**(1.00-**	**1.32)**
Kuchha	873/	101,441	55.8/	40.5	**1.64**	**(1.44-**	**1.87)**	1,077/	93,785	54.0/	41.1	**1.37**	**(1.23-**	**1.54)**
**Water**^**b**^														
Clean	1,208/	203,649	77.6/	81.5	1.00			1,596/	184,652	80.2/	81.2	1.00		
Not clean	348/	46,361	22.4/	18.5	**1.19**	**(1.05-**	**1.34)**	395/	42,879	19.8/	18.9	1.01	(0.90-	1.14)
**Latrine**^**c**^														
Inside - Dry Flush	174/	32,696	11.1/	13.1	1.00			165/	29,728	8.3/	13.1	1.00		
Inside - Flush	145/	46,188	9.3/	18.5	**0.71**	**(0.56-**	**0.90)**	144/	41,225	7.2/	18.1	**0.72**	**(0.58-**	**0.91)**
Outside	167/	30,301	10.7/	12.1	0.93	(0.75-	1.16)	216/	27,715	10.8/	12.2	**1.32**	**(1.07-**	**1.62)**
No latrine	1,079/	141,140	69.0/	56.4	1.14	(0.96-	1.35)	1,469/	129,194	73.7/	56.7	**1.74**	**(1.47-**	**2.06)**
**Fuel use**														
**Type of Fuel**														
Non-solid fuels^d^	204/	58,489	13.0/	23.3	1.00			230/	52,041	11.5/	22.8	1.00		
Solid fuels^e^														
Solid fuels^e^/Sep. kitchen	599/	99,665	38.2/	39.7	**1.22**	**(1.01-**	**1.48)**	756/	91,115	37.9/	39.9	**1.24**	**(1.04-**	**1.48)**
Solid fuels^e^/Non-sep kitchen	767/	92,834	48.9/	37.0	**1.43**	**(1.18-**	**1.73)**	1,010/	85,263	50.6/	37.3	**1.49**	**(1.24-**	**1.78)**
All^f^	1,366/	192,499	87.0/	76.7	**1.30**	**(1.08-**	**1.56)**	1,766/	176,378	88.5/	77.2	**1.33**	**(1.12-**	**1.58)**

* Estimated through Poisson regression, adjusted for age (in years), rural/urban, type of house, education of the head of the household, latrine type and number of siblings (as a continuous variable). ^a ^House types were categorized as follows: Pucca (high quality material - brick, wood - used for construction of roof, walls and floor), Semi-pucca (combination of high and low quality materials - mud thatch - in construction), and Kuchha (use of predominantly low quality materials). ^b ^Water was classified as clean when the major sources of drinking water were tap, hand-pump and tube-well/bore-well, as opposed to non-clean sources such as an open well, tank/pond, river/lake/spring. ^c ^Latrines were categorized as inside the house with a dry/chemical flush, inside the house with a regular flush, outside the house, and no latrine. ^d ^Non-solid fuels: kerosene, gas/natural gas, electricity and biogas; ^e ^Solid fuels: coal/coke/lignite, firewood, dung, and crop residue. ^f ^Exposed to solid fuels regardless of type of kitchen (estimated in a separated regression model).

**Figure 2:**
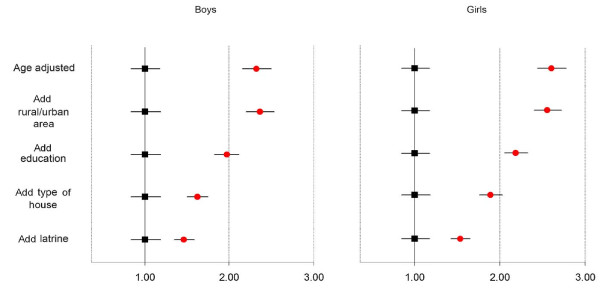
**Changes in Prevalence Ratio of solid-fuel**^**a **^**use among children that died between ages 1-4 years compared to living children of same age with stepped adjustment for other factors, India 1998-1999 **Footnote: ^a ^Solid fuels: coal/coke/lignite, firewood, dung, and crop residue. Confidence intervals estimated through floating absolute risks[38].

Deaths at ages 1-4 years had higher prevalence of solid-fuel use than living children of the same age (boys PR 1.30 95%CI 1.08-1.56; girls PR 1.33 95%CI 1.12-1.58). Solid-fuel use in non-separate kitchens was higher among boys that died at ages 1-4 years (PR 1.43 95%CI 1.18-1.73) than living boys of same age. Use of solid fuels in separate kitchens was also higher among boys that died at ages 1-4 years (PR 1.22 95%CI 1.01-1.48) compared to living boys of same age. Solid-fuel use was higher among girls that died at ages 1-4 years compared to living girls of same age (solid-fuel use in separate kitchens: PR 1.24 95%CI 1.04-1.48; solid-fuel use in non-separate kitchen: PR 1.49 95%CI 1.24-1.78). Tests for trend were significant regardless of gender (Table 1). When post-neonatal deaths were compared to living children below 1 year of age, the PR fell from 1.35 (95%CI 1.25-1.47 - data not shown) to 0.95 (95%CI 0.78-1.16) among boys and from 1.22 (95%CI 1.14-1.31 - data not shown) to 1.01 (95%CI 0.85-1.21) among girls after adjustment for rural/urban area, education of the head of the household, type of house, latrine, and number of siblings (Table 2). The prevalence ratio of solid-fuel use in non-separated kitchens fell from about 2.2 when adjusted for rural/urban area, education of the head of the household, type of house, and latrine to a prevalence ratio of about 1.4 for both genders (Figure 2).

**Table 2:** Prevalence Ratio of solid fuel use comparing post-neonatal deaths (between 29 days and 1 year of age) and living controls (younger than 1 year), India 1998-1999.

	Boys				Girls			
	**Post-Neonatal Deaths/Living Controls**				**Post-Neonatal Deaths/Living Controls**			
	**(n = 1,436)**	**(n = 68,529)**			**(n = 1,790)**	**(n = 61,873)**		
	**n**	**%**	PR*	**(95% CI)**	**n**	**%**	PR*	**(95% CI)**

**Type of Fuel**								
Non-solid fuels^a^	241/13,920	16.8/20.3	1.00		346/12,356	19.3/20.0	1.00	
Solid fuels^b^								
Solid fuels^b^/Sep. kitchen	584/28,008	40.6/40.9	0.95	(0.79-1.15)	640/25,316	35.8/40.9	0.85	(0.71-1.01)
Solid fuels^b^/Non-sep kitchen	613/26,630	42.6/38.8	0.95	(0.78-1.16)	804/24,224	44.9/39.1	1.01	(0.85-1.21)
All^c^	1,197/54,638	83.2/79.7	0.95	(0.79-1.15)	1,444/49,540	80.7/80.0	0.91	(0.77-1.07)

* Estimated through Poisson regression, adjusted for rural/urban, type of house, education of the head of the household, latrine type and number of siblings (as a continuous variable). ^a ^Non-solid fuels: kerosene, gas/natural gas, electricity and biogas; ^b^Solid fuels: coal/coke/lignite, firewood, dung, and crop residue. ^c ^Exposed to solid fuels regardless of type of kitchen (estimated in a separated regression model).

**Figure 3:**
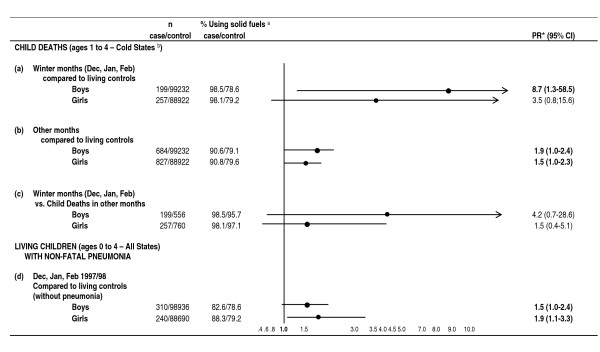
**Prevalence ratio of Solid Fuel **^**a **^**use among children that died in states with lower than average winter temperatures by month of death versus living controls and among children with reported pneumonia versus children without pneumonia **. Footnote: Cases are deaths that occurred during Dec, Jan, Feb and controls are deaths that occurred in the remaining months of the year in the same area. * Estimated through Poisson regression, adjusted for rural/urban, type of house, education of the head of the household, latrine type, number of siblings (as a continuous variable), and presence of smokers in the household. ^a ^Solid fuels: coal/coke/lignite, firewood, dung, and crop residue. Non-solid fuels: kerosene, gas/natural gas, electricity, and biogas; ^b ^Cold States or states with lower than average temperatures are: Arunachal Pradesh, Assam, Bihar/Jharkhand, Delhi, Chandigarh, Himachal Pradesh, Manipur, Meghalaya, Mizoram, Nagaland, Punjab, Sikkim, Tripura, and Uttar Pradesh/Uttarakhand.

In the colder states, during the winter months (December to February), the prevalence of solid-fuel use was slightly higher among children that died at ages 1-4 years (boys: 98.5; girls 98.1) compared to living children ages 1-4 years from the same areas (boys: 78.6; girls: 79.2), as well as when compared to child deaths at ages 1-4 years in the same areas during the other (non-winter) months (boys: 95.7; girls: 97.1) (Figure 3).

Prevalence of self-reported pneumonia among living children aged 0-4 years in the three months prior to the survey was 0.3% in colder states and 0.2% in warmer states. Overall, solid-fuel use among living children ages 0-4 years with pneumonia was higher than among living children ages 0-4 years without pneumonia (boys: PR 1.5 95%CI 1.0-2.4; girls: PR 1.9 95%CI 1.1-3.3, Figure 3).

Table 3 provides estimates of the number of deaths at ages 0-4 years in India that can be attributed to solid-fuel use, based on the total number of deaths estimated by the United Nations Population Division for 2004 [16,17]. No neonatal or post-neonatal deaths were attributed to solid-fuel use. Prevalence ratios for girls and boys ages 1-4 years were used to calculate deaths. About 128,000 of India's 2.4 million annual deaths (United Nations Population Division, Department of Economic and Social Affairs: World Population Prospects: The 2008 Revision) under age 5 can be attributed to solid-fuel use.

**Table 3:** Estimate of the excess number of child deaths (in thousands) associated with solid fuel use in India according to sex and age group, India 1998-1999.

	Boys		Girls	
	
Age group Type of Fuel	Attributable deaths/total deaths	%	Attributable deaths/total deaths	%
**Neonatal and Post neonatal**				
N/A (No deaths attributed to solid fuel use)	0/779	-	0/815	-
**1 to 4**				
Non-solid fuels ^a ^(a)	0/39	-	0/36	-
Solid fuels ^b^				
Solid fuels ^b^/Sep. kitchen (b)	21/114	18.0	23/118	19.4
Solid fuels ^b^/Non-Sep. kitchen (c)	44/146	30.1	52/157	32.9
All ^c ^(d)	60/259	23.1	68/257	24.8
TOTAL ATTRIBUTABLE DEATHS (a+b+c)	64/1077	6.0	75/1126	6.6
TOTAL ATTRIBUTABLE DEATH (a+d)	60/1077	5.6	68/1126	6.1

Note: PAF formula used (RR-1)/RR Total number of deaths from UN estimates for India in 2004 (United Nations Population Division, Department of Economic and Social Affairs: World Population Prospects: The 2008 Revision - File 4: Interpolated demographic indicators by major area, region and country, annually for 1950-2050 - Estimates, 1950-2010 - United Nations, Department of Economic and Social Affairs, Population Division (2009). World Population Prospects: The 2008 Revision, CD-ROM Edition.) ^a^Non-solid fuels: kerosene, gas/natural gas, electricity and biogas; ^b^Solid fuels: coal/coke/lignite, firewood, dung, and crop residue. ^c ^Exposed to solid fuels regardless of type of kitchen (estimated in a separated regression model).

## Discussion

### Child mortality risks from solid-fuel use

Deaths from all causes were 30% higher among children ages 1-4 years living in households where solid fuels were used. Child mortality from solid-fuel use in colder regions during winter months was even higher, probably as a consequence of the increase in time spent indoors and higher use of fuels for heating. In colder regions, solid-fuel use was also associated with self-reported pneumonia, especially among girls (Figure 3). Other studies show an association of solid fuel with pneumonia mortality [18] and respiratory symptoms among children under age 5 [19]. The observed modest risks appear to be consistent over time, as exposure to solid fuel has decreased only modestly (Figure 1). Moreover, we analyzed child mortality from solid-fuel use from the 2005-2006 National Family Health Survey [20] and found consistent associations (boys PR 1.60 95%CI 0.69-3.74 and girls PR 2.6 95%CI 1.18-6.03) but due to smaller sample size, these risks did not reach statistical significance (data available upon request).

Our observed risks for solid fuel use might still be explained by unmeasured differences in factors that are associated with solid-fuel use. The PRs for child deaths at ages 1-4 years compared to living children of the same age fell considerably after adjustment for child's age, rural/urban area, education of the head of the household, type of house, access to latrine and number of siblings (Figure 2).

For example, we could not measure a child's nutritional status, and this is an important factor contributing to child mortality from respiratory diseases [21]. On the other hand, not attributing neonatal or post neonatal deaths to solid-fuel use may underestimates our absolute mortality projections since solid-fuel use may result in pre-term deliveries and low birth weight, although such increases have not been quantified [9].

### Gender differences

With the exception of the colder regions of India, girl deaths at ages 1-4 years had higher prevalence of solid-fuel use when compared to living girls of the same age than did boys. The frequency of self-reported pneumonia was not different between boys and girls but we observed higher solid-fuel use among girls with self-reported pneumonia. Time spent indoors and proximity to pollution source influence the level of exposure[9] and data also suggest that girls spend more time indoors than boys [22]. Other factors such as gender preference [23] may also influence treatment access and we believe that differential access to health services may explain the higher mortality among girls. Indeed, hospital based studies have shown that boys are more likely than girls to be admitted for acute respiratory infections [24]. Such findings are sometimes interpreted as if boys had a higher susceptibility to respiratory infections than girls, but the higher hospital admission rates for boys may be a consequence of the gender differences in access to health care [25-29]. Treating child respiratory symptoms from solid fuel smoke may prevent child deaths while not affecting the incidence of respiratory symptoms, and indeed boys and girls had equal incidence of self-reported pneumonia but girls had higher mortality [30]. Other factors may explain the gender differences in survival after the onset of pneumonia and further studies are needed to clarify the role of severity of respiratory diseases and other biological factors (i.e. ability to overcome disease/survive) [31,32]. Our study is less subject to gender-based selection bias because it collects information directly in the household.

### **Absolute number of child deaths**

The large proportion of children exposed to solid-fuel use in India explains why, even in the presence of lower risk estimates than made previously, solid-fuel use is still responsible for one quarter of all deaths at ages 1-4 years, or 6% of all deaths under age 5 (Table 3). Our prevalence ratios and absolute totals (128 000) are lower than the earlier indirect estimates of child deaths from solid-fuel use in India from the Global Burden of Disease (GBD), which range from 161 000 to 261 000 [2,15]. But these earlier estimates are quite uncertain for two reasons. First, the ratio of lower respiratory infections to total child mortality in the GBD varies from 22.9% in the 2001 version [2] to 14.1% in the 2004 [15]. Second, the attributable fraction for solid fuels for lower respiratory infections is 52% in the GBD, but this is based on small, non-representative studies in South Asia, with inadequate adjustment for other household exposures, such as access to water and sanitation. Moreover, the GBD risk estimates relied on studies that estimated odds ratios, which tend to overestimate the true relative risk when exposure is common, as is the case with solid fuel use [14]. Direct estimates of child mortality in India [33] suggest that lower respiratory infections are responsible for 19.6% (18.4% for boys and 21.0% for girls) of all child deaths, or 432 000 of all 2.2 million child deaths in India in 2004 [2,9,15]. One of the major limitations of the study is the fact that we did not have information on cause-specific mortality in this study. However, if we assume that solid-fuel use results in deaths only from acute respiratory infections, then we would attribute about 30% (128 000/432 000) of all child deaths from lower respiratory infections to solid-fuel use.

## Conclusion

Solid-fuel use accounts for fewer child deaths than suggested by earlier indirect estimates. Still, it may be responsible for about 6% of all deaths at ages 0-4 years, about 20% of deaths at ages 1-4 years, or about 128 000 child deaths a year in India. The high proportion of the population exposed to solid-fuel, but lower than previously estimated relative risks [18,30,34], account for the large absolute number of child deaths. Solid fuel use has declined only modestly in the last decade. This suggests that marked reductions in child mortality from solid-fuel use might only arise with more rapid economic development [9] spurring far more households to use clean fuel sources [35,36]. However, while declines in solid fuel use in the future are uncertain, complementary strategies exist today that would reduce child mortality from acute respiratory infection. These include expanded case management for pneumonia and introduction of newer antigens such as those against haemophillus B and streptococcus pneumonia into child vaccination programs (the latter of which might also reduce gender inequalities in child mortality) [21,37].

## Competing interests

The authors declare that they have no competing interests.

## Authors' contributions

PJ and the academic partners in India (RGI-CGHR Collaborators) have planned the study in close collaboration with the Office of the Registrar General of India (RGI). DGB and PJ conducted the statistical analyses. All authors participated in interpreting the data and writing the manuscript and have read and approved the final manuscript.

## Pre-publication history

The pre-publication history for this paper can be accessed here:

http://www.biomedcentral.com/1471-2458/10/491/prepub
